# Cyclic mechanical stimulation of TDCs improves tendon tissue maturation *in vitro* by enhancing collagen formation

**DOI:** 10.3389/fbioe.2026.1773418

**Published:** 2026-07-23

**Authors:** Ekaterina A. Oleinik, Felix Groß, Magdalena Fuchs, Lune Teplitchi-Menou, George Gourgi, Büsra Külekci, Andreas H. Teuschl-Woller, Philipp J. Thurner

**Affiliations:** 1 Institute of Lightweight Design and Structural Biomechanics, TU Wien, Vienna, Austria; 2 Department Life Science Engineering, UAS Technikum Wien, Vienna, Austria; 3 Austrian Cluster for Tissue Regeneration, Vienna, Austria

**Keywords:** 3D cell culture, bioreactor, maturation, mechanical stimulation, tendon tissue engineering

## Abstract

Tendinopathy represent a growing clinical problem and unmet therapeutic need, mainly due to the limited intrinsic regenerative capacity of tendons and our poor understanding of the underlying disease mechanisms. A major obstacle to progress in treatment is the lack of physiologically relevant models that accurately reflect the structure, mechanics and biology of tendons. While animal models provide valuable insights into tendon pathology and repair, *in vitro* 3D models offer a controlled and ethical alternative for studying the pathogenesis and progression of tendinopathy, especially investigating specific mechanobiological mechanisms. However, the lack of a standardized *in vitro* model makes it difficult to compare results from different studies, particularly with respect to the mechanical stimulation regimes applied. Existing approaches use either static or cyclic mechanical loading, and there is a lack of scientific consensus over which approach is advantageous. In this study, we addressed this question using a custom-made displacement-controlled bioreactor to compare the effect of static vs. cyclic loading on the development of 3D tissue-engineered tendons. In our experiments, cyclic loading application resulted in increased expression of tendon maturation markers, namely *Col1, Tnmd,* and *Sparc*, and cell elongation and alignment along the strain axis compared to static loading. Additionally, we report that cyclic loading promotes cell survival, proliferation and matrix remodeling, with enhanced collagen deposition. Structural and mechanical characterization confirmed fibril formation upon cyclic loading and higher stiffness of cyclically stimulated constructs. Together, these findings indicate that cyclic mechanical stimulation promotes key biological, structural, and mechanical features of early-stage tendon tissue maturation and provides a more favorable microenvironment for the development of *in vitro* tendon models compared to static loading.

## 1 Introduction

Tendinopathies are serious musculoskeletal diseases caused by overload or microtrauma (Abate et al., 2009; Fu et al., 2010). They are characterized by localized activity-related pain, tendon swelling and altered tissue biomechanics, resulting in limited mobility, and in severe cases, complete loss of tendon function (Millar et al., 2021; Lipman et al., 2018; Gehwolf et al., 2025). The number of tendinopathy cases is increasing, not only among athletes, creating a growing clinical and socio-economic burden worldwide (Lipman et al., 2018). Effective treatment remains challenging due to the limited regenerative capacity of tendon tissue and its highly specialized extracellular matrix (ECM) organization. Currently, clinical management frequently involves a choice between a conservative symptomatic treatment to dampen pain and inflammation or surgical intervention in more advanced cases. As a result, substantial research efforts are currently focused on tendon regeneration and repair. Accumulating evidence supports the beneficial effects of physiotherapy-based interventions, including extracorporeal shock wave therapy (Gehwolf et al., 2025; Irby et al., 2020), and eccentric exercises (Alfredson et al., 1998; Krogh et al., 2022; Radovanović et al., 2023; Traweger et al., 2025), with the last showing the best outcomes in promoting tendon healing, and these approaches are widely implemented in clinical practice. Although many patients benefit from structured loading-based rehabilitation, these interventions are often prolonged, demanding for both patients and clinicians, and full restoration of native tendon structure and mechanical properties is not consistently achieved.

Tendon healing involves matrix remodeling processes that may result in altered collagen organization leading to the formation of mechanically inferior scar tissue, which compromises structural integrity, impairs functional restoration, and increases the risk of re-injury (Irby et al., 2020; Riley, 2008; Loiacono et al., 2019). These limitations underscore the urgent need for a more comprehensive understanding of the pathophysiological mechanisms underlying tendinopathy, and the mechanisms of tissue adaptation to it (Gehwolf et al., 2025; Gaut and Duprez, 2016; Andarawis-Puri et al., 2015), thereby enabling the development of more targeted and effective therapeutic strategies. For that, physiologically relevant experimental models that can accurately reproduce structural, mechanical and biological characteristics of natural tendons are needed (Meeremans et al., 2021).

While *in vivo* models, such as rodents or horses (Lake et al., 2008; Luo et al., 2023; Zhang et al., 2022; Wang et al., 2021; Vidal et al., 2024) remain indispensable for understanding systemic regenerative responses and have provided valuable insights into the mechanisms of tendon injury, inflammation and healing, their widespread use is limited due to ethical considerations, as well as our inability to attribute the observed beneficial effects to specific experimental parameters, as systemic factors (i.e. inflammation, vascularization, endocrine signaling) can significantly affect the outcomes. In contrast, *in vitro* systems, while not fully recapitulating the complexity of the physiological tendon environment, provide a powerful experimental platform. By allowing precise and independent control of cellular, biochemical, and mechanical parameters, such as loading mode, strain amplitude, frequency and duration, they enable the establishment of a highly defined and reproducible experimental environment. This level of control enables systematic interrogation of load-dependent cell behaviour and ECM remodeling that cannot be achieved *in vivo*, thereby providing mechanistic insights that complement animal models (Theodossiou and Schiele, 2019; Gomez-Florit et al., 2022; Benage et al., 2022; Wunderli et al., 2020; Citeroni et al., 2020; Wang et al., 2018a).

Notably, to date there is still no standardized *in vitro* model of tendon that mimics the hierarchical organization and viscoelastic properties of the collagen-rich tendon matrix, which in turn is required to promote effective mechanotransduction and coordinated ECM remodeling in response to injury.

Mechanical loading is one of the key players in the development, maintenance and healing of tendons (Andarawis-Puri et al., 2015; Scott et al., 2011; Schiele et al., 2013; Galloway et al., 2013; He et al., 2022; Killian et al., 2012). Physiological levels of strain (up to 10%) stimulate tenocyte alignment, proliferation and ECM synthesis, whereas overloading or mechanical deprivation can lead to inflammation, degeneration and fibrosis (Xu et al., 2023; Chen et al., 2024; Spiesz et al., 2015; Thorpe et al., 2015; Screen et al., 2005; Huisman et al., 2014; Kjær et al., 2009; Magnusson and Kjaer, 2019). Therefore, to model healthy tendon tissue *in vitro,* it is crucial to reproduce the appropriate mechanical cues. Despite significant advances in this area, there is no consensus on optimal mechanical stimulation protocols, and currently both static and cyclic loading are used with varying, in some cases even contradictory, results (Sheng et al., 2020; Gögele et al., 2025). While static loading is technically straightforward, it often relies on the stresses generated by cell contraction, e.g. within pinned constructs, which provide limited control over strain or stress magnitude (Gehwolf et al., 2019). In contrast, cyclic loading mimics physiological conditions of tendons more closely but requires specialized bioreactor systems that can deliver reproducible and controlled stimulation (Tomasch et al., 2023; Burk et al., 2016; Kataoka et al., 2020).

We hypothesize that cyclic mechanical loading provides a more favorable environment for tendon-like tissue formation *in vitro* compared with static loading, promoting enhanced ECM remodeling and functional maturation, ultimately resulting in superior mechanical properties of engineered tendon constructs.

Here, to directly address this gap and systematically evaluate the influence of loading mode on tendon tissue formation, we used a custom-designed, displacement-controlled bioreactor (MagneTissue (Heher et al., 2015)) to directly compare the effects of static (SL) and cyclic (CL) mechanical loading on fibrin-based 3D tendon constructs. To recreate the key structural and biochemical characteristics of natural tendon tissue, we combined the three key components of the classic tissue engineering triad approach (Akter, 2016). We used rat tendon-derived cells (TDCs), due to their intrinsic tenogenic potential, the ability to retain the phenotypic characteristics of native tenocytes and their pronounced responsiveness to mechanical and biochemical signals (Ansorge et al., 2011; Docheva et al., 2005; Ruiz-Alonso et al., 2021; Zhang et al., 2019; Lui and Chan, 2011). These cells were embedded in fibrin, a biocompatible, biofunctional scaffold material with a porous architecture promoting cell attachment, to provide a temporary but physiologically relevant matrix for tissue regeneration (Li et al., 2024; Brown and Barker, 2014; Breidenbach et al., 2015), which has found wide applications in the field of tissue engineering (Heher et al., 2015; Tomasch et al., 2022; Ciardulli et al., 2024; Hromada et al., 2024).

By combining histological, mechanical and transcriptomic analyses, this study aimed to identify the biological and structural differences that occur in tendon development *in vitro* in response to mechanical loading. Through a systematic comparison of static and cyclic loading, we address a key methodological question in tendon tissue engineering and supports the development of standardized loading protocols (Thorpe et al., 2015) for *in vitro* tendon modeling.

With our results, we showed that cyclic loading promotes coordinated tenocyte alignment, upregulation of tendon-associated gene expression, organized extracellular matrix deposition, and enhanced functional maturation compared with static loading conditions.

## 2 Methods

### 2.1 Tenocyte isolation

Primary TDCs were isolated from dissected Achilles tendons of male Sprague Dawley rats as previously described (Gehwolf et al., 2019). Briefly, isolated tendons (1 donor, both hind limbs) were cut into small pieces and enzymatically digested using 3 mg/ml collagenase Type II (Gibco, USA) at 37 °C and 5% CO_2_ overnight in complete medium (Dulbecco’s Modified Eagle’s Medium High Glucose (DMEM HG) (VWR, Belgium), 10% (v/v) fetal bovine serum (FBS) (Cytiva-HyClone Laboratories, USA), 1% L-glutamine 100X (VWR, Belgium) and 1% Penicillin/Streptomycin 100X (P/S) (Biowest, France)). The next day, tissue was further dissociated by trituration and filtered through a 70 µm cell strainer. The cell suspension was centrifuged at 200 g for 5 min. TDCs were then seeded in complete media onto a T25 flask. Media was exchanged every second day, and the cells were passaged at approximately 70% confluency. Cells in passages 5-6 were used for the experiments.

### 2.2 Fibrin ring fabrication

**FIGURE 1:**
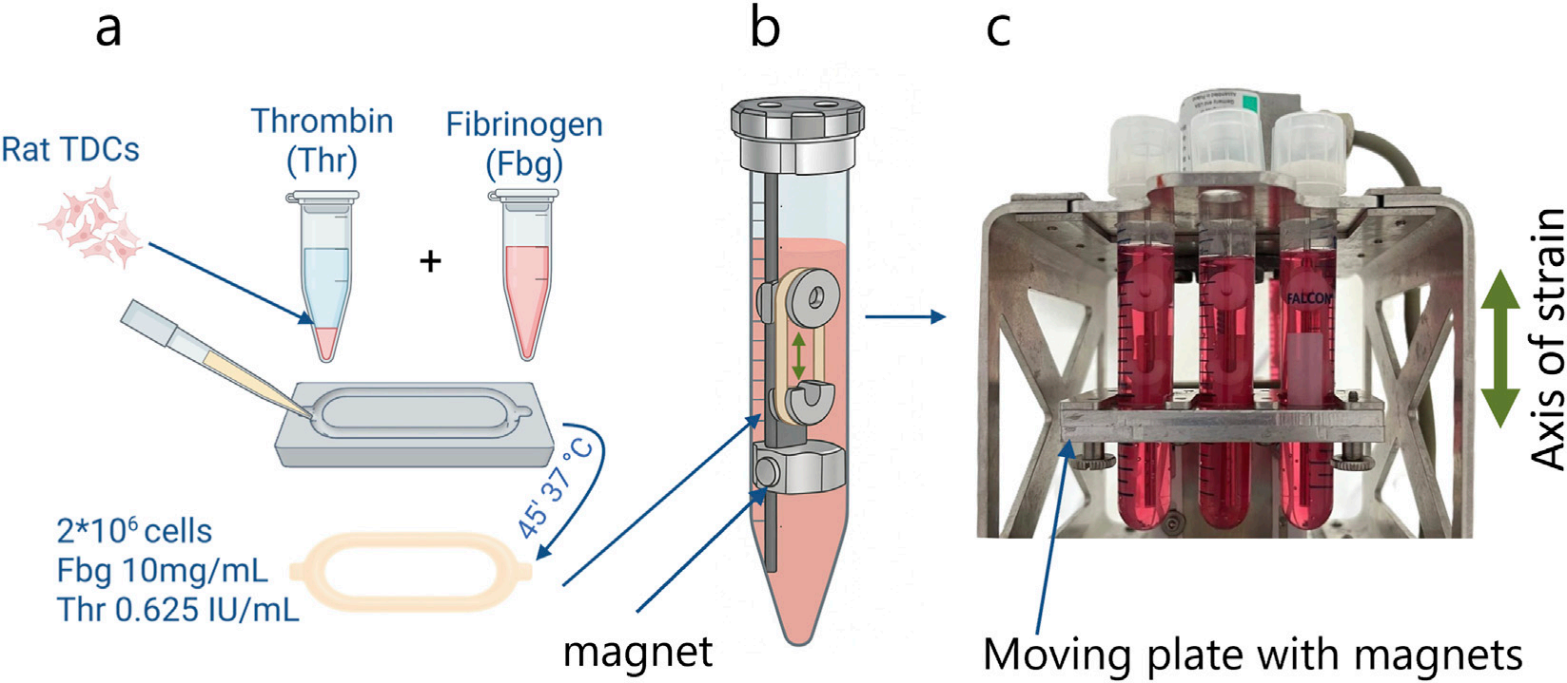
Experimental setup of tendon constructs preparation using MagneTissue bioreactor. **(a)** Rat tendon-derived cells (TDCs) at a concentration of 4x10^6^ cells/mL were embedded in ring-shaped fibrin scaffolds with final concentrations of fibrinogen=10 mg/mL and thrombin=0.625 IU/mL, and ring geometry parameters: 3 mm height, 2 mm in strand diameter. **(b)** Schematic view of a sample mounted between spool and hook, which has a magnet attached to it. **(c)** Custom-made bioreactor system, called MagneTissue, was used for the controlled mechanical stimulation of tendon constructs by vertical movement of the hooks, achieved through magnetic forces between hooks and moving plates.

TDCs were embedded in ring-shaped fibrin hydrogels using the clinically approved Tissucol Duo 500 5.0 mL Fibrin Sealant (Baxter AG, Austria) as previously described (Tomasch et al., 2022). Briefly, fibrinogen was diluted to a concentration of 20 mg/mL with DMEM HG w/0 serum. Thrombin was first diluted to 4 IU/mL with 40 mM CaCl_2_ in two steps (Thrombin 500 (stock concentration)-Thrombin 50-Thrombin 4), and then the cell pellet was diluted with DMEM-HG w/0 serum and Thrombin 4 IU/mL to a final Thrombin concentration of 1.25 IU/mL. To cast ring-shaped hydrogels, 250 µL of diluted fibrinogen was mixed with 250 µL thrombin/cell suspension, gently mixed, injected into silicon scaffold molds and polymerized at 37 °C and 5% CO_2_ for 45 min (Figure 1a). The ring-shaped scaffolds had a final concentration of 2 × 10^6^ TDCs in 10 mg/mL fibrin and 0.625 IU/mL thrombin. The ring dimensions are 3 mm in height and 2 mm in strand diameter.

In this study, the concentrations of fibrin and thrombin were chosen and adapted based on previous studies on skeletal muscle (Tomasch et al., 2022) and Schwann cells (Tomasch et al., 2023) and our own preliminary results with tendon-derived cells (TDCs).

### 2.3 MagneTissue bioreactor

MagneTissue bioreactor is a custom-made bioreactor system that exerts uniaxial tensile loading on ring-shaped scaffolds (Heher et al., 2015). To accomplish this, the scaffolds were mounted between two attachments, the so-called spool (upper attachment) and hook (lower attachment), that are attached to specific inserts designed to fit into 14 mL Snap-Cap Falcon tubes (Corning Science, Mexico) (Figure 1b). The hooks contain magnets that interact with magnets embedded in the bioreactor plates (Figure 1c). Vertical movement of the hooks is achieved through magnetic forces between the hooks and plates, thus translating vertical plate movement in a contactless manner. As a consequence, the ring-shaped scaffolds are stretched, ideally leading to uniform tensile stress in the linear region of the sample. The movement of the plates is realized by a stepper motor controlled by a microcontroller. The MagneTissue bioreactor has two working modes: the static mode, when the magnetic plate moves to a prescribed position and stays there for a prescribed time (from seconds to hours), and the cyclic mode, where the plate moves up and down in a linear trapezoidal ramp motion to a prescribed position at a specified frequency (up to 1.2 Hz) for a prescribed time.

### 2.4 Mechanical stimulation

**FIGURE 2:**
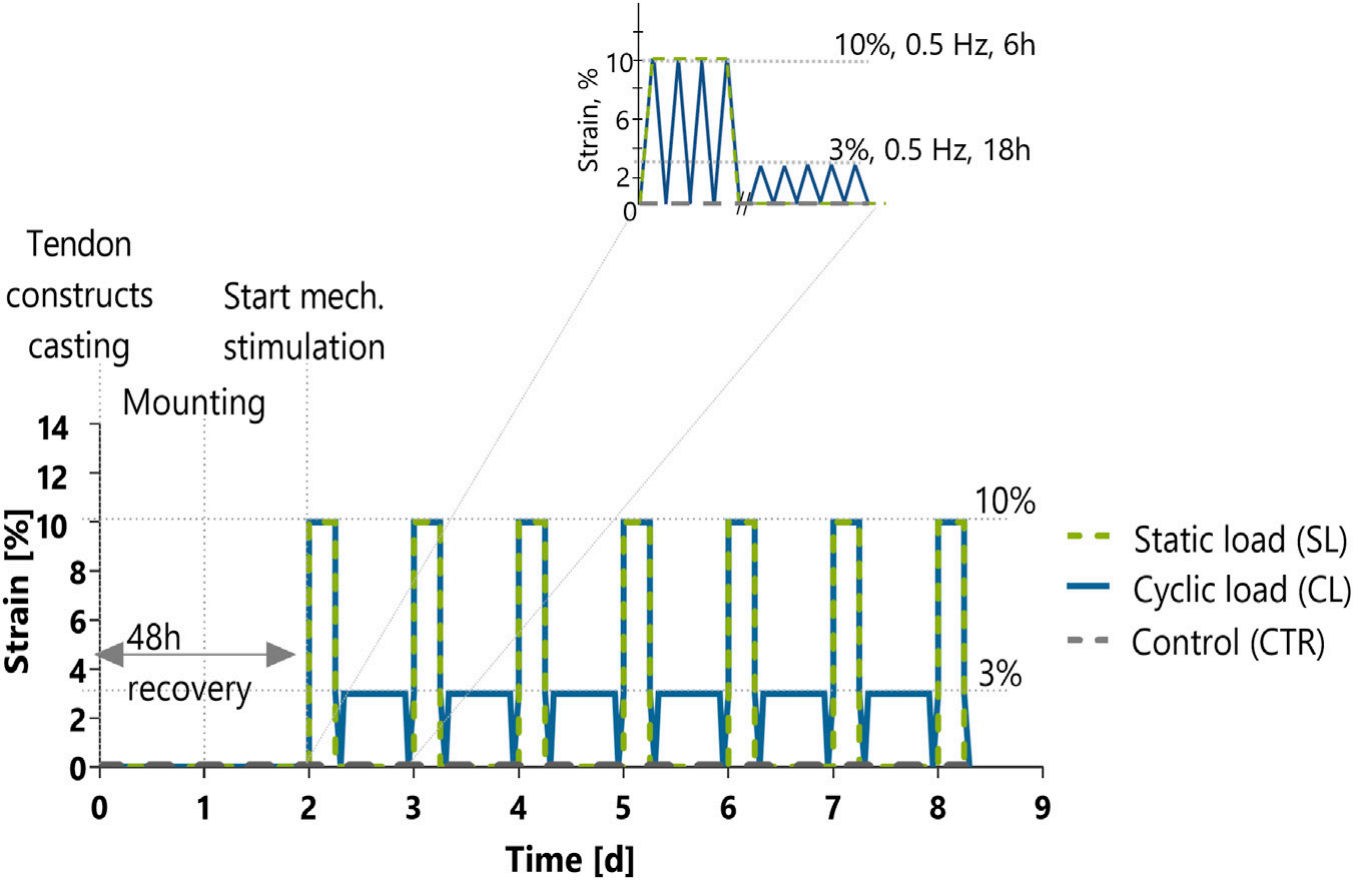
Mechanical stimulation protocol using MagneTissue bioreactor. We applied 10% uniaxial tensile strain for 6 h daily, followed by an 18-h resting period (at zero position), over 7 consecutive days. Two loading modes were compared: static (SL) and cyclic loading (CL). CL was applied with a 0.5 Hz frequency, with a 3% strain maintained during the 18-h resting period to ensure constant media agitation.

Tendon constructs were cultured in the complete media supplemented with 100 KIU of aprotinin (serine protease inhibitor used to reduce the risk of fibrin digestion (Pentapharm, Switzerland)), 0.2 mM ascorbic acid (2-Phospho-L-ascorbic acid trisodium salt) (Sigma-Aldrich, USA) and 0.1 mM L-proline (Sigma-Aldrich, USA), which are important for collagen synthesis (Murad et al., 1981; Russell et al., 1981; Fisher et al., 1991; Karna et al., 2020) without mechanical stimulation for 2 days for cell recovery. Then, samples were transferred to the bioreactor and subjected to tensile load in two different modes: static (SL) and cyclic (CL) loading. In both modes, samples were pulled to 10% tensile strain (3 mm displacement) for 6 h per day, followed by a rest period of 18 h per day for 7 consecutive days. In case of CL, all strains were applied in a dynamic fashion at a frequency of 0.5 Hz with a 3% strain maintained during the 18-h resting period to ensure media agitation. The loading protocols are graphically summarized in Figure 2. Constructs, suspended between the spool and the hook without any load application, were used as a control group and called CTR. In the present study, static loading refers to the application of combined initial constant external strain and subsequent cell-mediated tension due to fibrin relaxation and anchorage between the spool and the hook.

The mechanical stimulation regimes were chosen based on our own preliminary experiments, literature analysis and the conditions encountered *in vivo* during embryonic development and rehabilitation. In addition, the applied frequency (0.5 Hz) and strain amplitude of the cell-laden scaffolds (10%) are both within the physiological range of tendons and were also used in several previous studies (Sheng et al., 2020; Burk et al., 2016; Tomasch et al., 2022).

### 2.5 RNA isolation

Total RNA was isolated from one-half of the ring (N=6 for each group for each experiment(n=3)) using the protocol established by Hromada et al. (2024). Briefly, total RNA was extracted using innuSOLV RNA Reagent (IST Innuscreen GmbH, Germany) and Precellys® tissue homogenizer (Bertin Technologies SAS, France). Samples were first washed with PBS, transferred to Precellys tubes containing zirconium oxide beads with innuSOLV RNA Reagent, and snap-frozen in liquid nitrogen. After thawing, samples were homogenized in three cycles (5500 rpm, 20 s each) and centrifuged at 15,800 g to remove fibrin residues. RNA was isolated using a two-step chloroform extraction, followed by ethanol precipitation. The aqueous phase was mixed with 70% ethanol, incubated, and centrifuged to pellet RNA. The RNA pellet was washed three times with cold ethanol, air-dried, and resuspended in nuclease-free water. The RNA quality and quantity were assessed using a Nanodrop One C spectrophotometer. All centrifugation steps were performed at 4 °C.

### 2.6 cDNA synthesis, PCR analysis

For cDNA synthesis, RNA was reverse transcribed using the FIREScript Reverse Transcriptase kit (Solid Biodyne, Estonia), following the manufacturer’s protocol. Per sample, 5 μM of random primers were used. The reaction was equilibrated for 10 min at 25 °C, followed by reverse transcription at 50 °C for 7 min, and enzyme inactivation at 85 °C for 5 min. The resulting cDNA was diluted to a concentration of 3.3 ng/μL in RNase-free water, and RT-qPCR was performed using 3 µL of the diluted cDNA template, 5 µL of SYBR Green FAST qPCR Master Mix, and 200 nM of each primer (forward and reverse), in a total reaction volume of 10 µL (primer sequences listed in Table 1). Reactions were run on a qTOWER3 G Real-Time PCR Detection System (Analytik Jena, Germany). Thermal profile included initial denaturation for 10 min at 95 °C, 40 cycles of 20 s at 95 °C, 20 s at 58,3 °C (*MMP3*), 59,5 °C (*Tnc, Tnmd, Scx*), 60 °C (*Col1, Col3, Gapdh*), 64 °C (*Sparc*) and 66 °C (*Loxl1*), 20 s at 72 °C, followed by a melting curve. All samples were analyzed in triplicate. Target gene expression was calculated with the comparative Ct (ΔΔCt) method and *Gapdh* was used as a housekeeping gene.

**TABLE 1:** RT-qPCR details for primers used in this study.

Gene Symbol	Primer Sequence (5′–3′)	Annealing Temp [°C]	Thermal profile
*Scx*	F: CCA​CTC​CAG​TCC​GAA​CAC​AT	59.5	Fast-2-step
	R: TCA​TGC​CGC​CTC​TTT​AGG​TC		
*Tnc*	F: ATG​TTC​CAA​AGA​GCC​AGC​AA	59.5	Fast-2-step
	R: AGG​CTG​TAG​TTG​AGG​CGG​TA		
*Tnmd*	F: GGC​CCG​AGG​TAT​CCA​AGA​AG	59.5	Fast-3-step
	R: AGA​TGC​CAG​TGT​ATC​CGT​TTT​T		
*Sparc*	F: TGG​CAA​GGT​GTG​TGA​GCT​GGA​C	64	Fast-2-step
	R: GGA​GCT​TGT​GGC​CCT​TCT​TGG​T		
*Col1A1*	F: CGC​AGG​AAG​GTC​AGC​TGG​ATA​G	60	Fast-3-step
	R: GCA​GGA​AGG​TCA​GCT​GGA​TAG		
*Col3A1*	F: TGG​GAT​CCA​ATG​AGG​GAG​A	60	Fast-3-step
	R: TCATGGCCTTGCGTGTTT		
*MMP3*	F: TCC​AAA​TGC​AGG​GAA​AGT​GAC​CCA	58.3	Fast-2-step
	R: GCG​CCA​AGT​TTC​AGA​GGA​AGA​GC		
*Loxl1*	F: CAT​TTT​CCC​CTG​GCT​TCT​CCG​C	66	Fast-2-step
	R: CCA​AAA​GGG​ATC​TCC​ATC​CTT​GTC​C		
*Gapdh*	F: TCT​CTG​CTC​CTC​CCT​GTT​CTA	60	Fast-3-step
	R: CGA​TAC​GGC​CAA​ATC​CGT​TC		

### 2.7 RNA sequencing

Oxford Nanopore Technology (ONT) sequencing was performed using the MinION Mk1d device (Oxford Nanopore Technologies, United Kingdom) following the manufacturer’s guidelines. Strand-switching reverse transcription using cDNA-PCR Barcoding Kit SQK-PCB114.24 (Oxford Nanopore Technologies, United Kingdom) was performed to ensure full-length cDNA synthesis and compatibility with downstream library preparation. Total RNA (650 ng for each sample) was first subjected to reverse transcription to generate cDNA. After reverse transcription, cDNA was PCR-amplified to obtain sufficient input material for sequencing. The resulting cDNA underwent end-repair and dA-tailing to create blunt, adenylated ends suitable for adapter ligation. ONT adapters (barcodes) were then ligated to the cDNA for each sample specifically. In the end, a bead-based cleanup was performed to remove enzymes, salts, primers, and short cDNA fragments, ensuring library purity and optimal sequencing performance. Finally, prepared libraries were quantified using Qubit flex fluorometer (Thermo Fisher Scientific, USA), and their integrity was evaluated using an Agilent Tapestation 4150 (Agilent Technologies, USA).

50 fmol of cDNA was pooled and loaded onto a pre-conditioned R10.4.1 flow cell (Oxford Nanopore Technologies, Oxford, United Kingdom), primed with ONT sequencing buffer and loading beads, before sequencing on MinION. The sequencing run was initiated using MinKNOW software v24.11.10 (Oxford Nanopore Technologies, United Kingdom), with real-time basecalling performed using the Guppy (Dorado v7.6.8) basecaller. The run was monitored continuously to assess data quality. Following sequencing, raw FAST5 files were converted to FASTQ format and subjected to quality control using FastQC. Following adapter trimming using cutadapt (Martin, 2011) and quality filtering (Q>=7.0), reads for each sample were successfully aligned to the reference genome Rattus_norvegius.mRatBN7.2 using the minimap2 alignment tool (Li, 2018).

Data were further processed using a custom RStudio pipeline, which included principal component analysis (PCA) to assess sample clustering and analysis of differential gene expression using the DESeq2 package (version 1.46.0) following standard processing as described by *Love et al.* (Love et al., 2014). The results table was converted to a tibble using the tidyverse package (Wickham et al., 2019) and merged with *Rattus norvegicus* gene annotations retrieved from Ensembl BioMart (Carlson, 2026). Ensembl gene identifiers were mapped to external gene names using the BioMart package and gene symbols were extracted for enrichment analysis.

Gene ontology (GO) Biological Process enrichment was performed using clusterProfiler with the org.Rn.eg.db genome-wide annotation database for the genes with padj>0.1((Yu et al., 2012)). Enrichment network visualisation was generated with enrichplot, displaying the top 20 enriched categories (Enrichplot, 2025).

**FIGURE 3:**
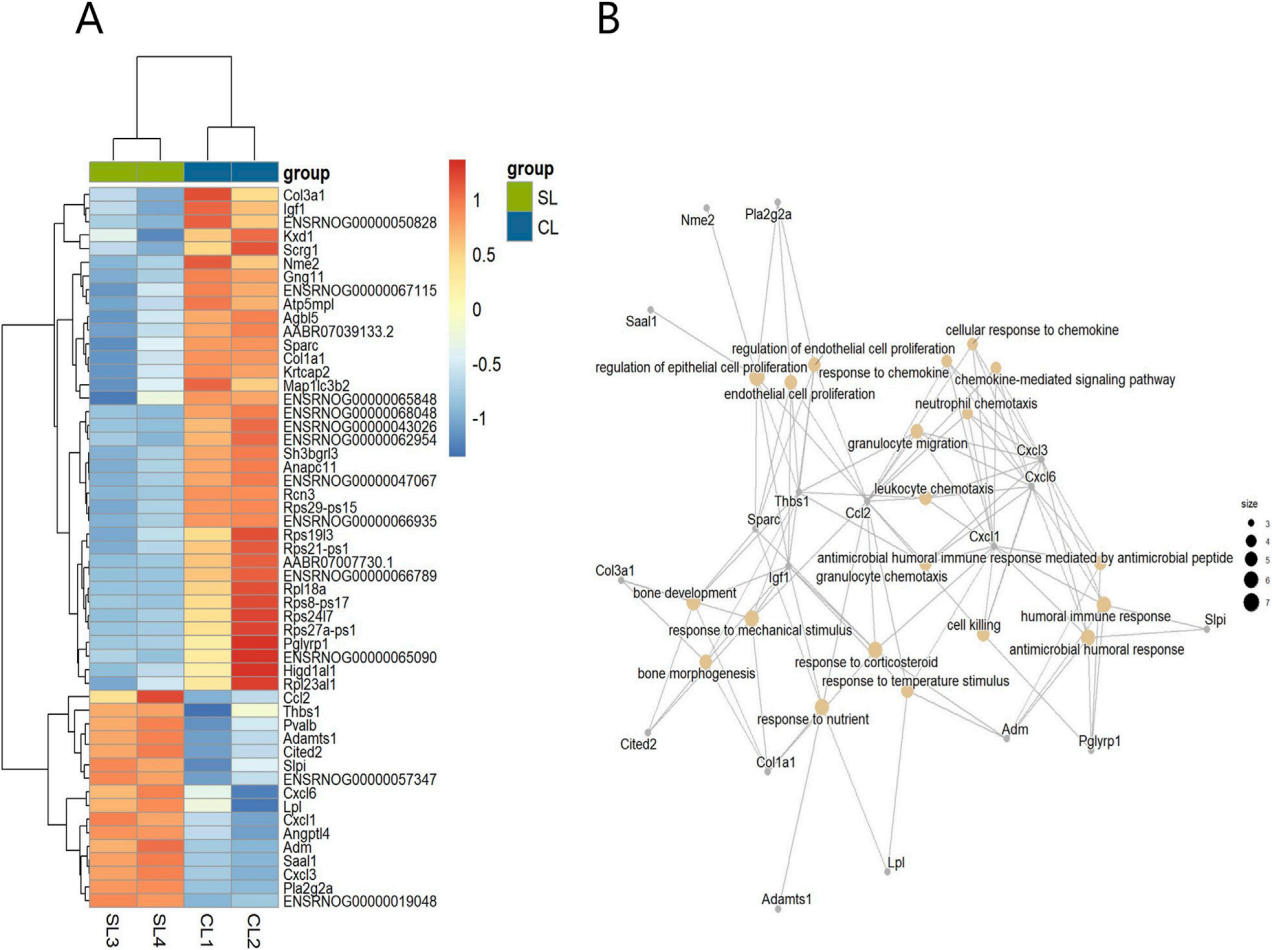
Transcriptomic profiling of TDCs subjected to mechanical stimulation in MagneTissue Bioreactor. Two loading modes were compared: SL and CL. **(A)** Heat-map, illustrating the expression profiles of significantly differentially expressed genes (DEGs) identified by ONT RNA-seq analysis between CL and SL groups. Gene expression values are shown as normalized and row-scaled z-Scores across samples. Upregulated and downregulated genes are indicated in red and blue, respectively. **(B)** Functional enrichment and pathway clustering of DEGs based on Gene Ontology (GO) analysis. Enriched pathways are grouped by biological process, with bubble size representing the number of genes.

RNA sequencing was performed in two separate runs due to technical limitations of the loading system (flow cell FLO-MIN114) and the cDNA yield, with each run containing two CL and two SL samples, where each sample represented pooled 3 independent replicates from 2 experimental runs. In total, 4 samples from the CL group and 4 samples from the SL group were analyzed. The data shown in Figure 3A correspond to one sequencing run, while the second run was used to verify the consistency of the observed trends. In the Supplementary Figure S4 (Supplementary Materials) the results of both runs are depicted.

### 2.8 Histology staining

For each independent experiment (n=3), six samples were collected for histological analysis. For that, one half of the ring (linear region) was fixed in 4% Histofix for 3 h at RT, embedded in paraffin using a TP1020 Automatic Tissue Processor (Leica Microsystems, Germany), and then longitudinally sectioned using a Histocore Arcadia C microtome (Leica Microsystems, Germany). The cut sections (10 μm) were further processed according to standard histological or immunostaining protocols as described below.

#### 2.8.1 Martius, Scarlet and Blue (MSB) staining

To analyse ECM remodeling and differentiate between fibrin and collagen, MSB staining (Sanova Pharma GesmbH, Austria) was performed according to the manufacturer’s protocol. Briefly, sections were first stained with Martius yellow solution for 3 min to detect early fibrin deposition, followed by rinsing in distilled water. The sections were then stained with Crystal Ponceau solution for 10 min to visualize mature fibrin and subsequently differentiated in phosphomolybdic acid for 5 min to remove excess dye from collagen and cytoplasm. Following this, sections were stained with Methyl Blue solution for 2 min, followed by an acetic acid washing step to visualize collagen fibers. After the final staining step, the sections were dehydrated in ascending concentrations of ethanol, cleared in xylene, and mounted with resin-based mounting medium.

Collagen deposition was quantified by Colour deconvolution2 plugin (Ruifrok-Johnston method) using the Masson/Trichrome vectors in Fiji to isolate the collagen (blue) optical-density image (Ruifrok and Johnston 2001). Three non-overlapping regions of interest (ROIs) were selected for each sample. Within each ROI, 5-7 measurements were obtained on each side (left and right) using Fiji Straight Line tool. Thickness was defined as the shortest perpendicular distance from the external surface to the inner boundary of the collagen-positive layer. The obtained measurements were averaged to yield a per-ROI value; sample thickness was calculated as the average of the three region of interest values. All image-processing settings were held constant across samples to ensure comparability.

#### 2.8.2 Actin-FITC staining

Sections were dewaxed and dehydrated using an Xylol-Ethanol cassette. Before the antibody incubation, we performed the antigen retrieval step using a Hot citric buffer for 20 min. Then sections were treated with a permeabilization buffer (PBS + 0.25% Triton X100 (PBST)), followed by a blocking step (PBST + 5% Goat Serum (GS)) and incubated overnight in a humid chamber at +4 °C with primary anti-β-Actin–FITC antibody (1:200, F3022, Sigma Aldrich) diluted in 0.1% BSA + 1% GS in PBST, followed by cell nuclei counterstaining with 4’,6-diamino-2-phenylindole (DAPI) diluted in 1:2000 for 15 min before mounting.

#### 2.8.3 EdU click-chemistry assay

Analysis of cell proliferation was performed using the EdU-Click Chemistry 488 kit (BCK-EDU488, Sigma-Aldrich) according to the manufacturer’s instructions. Briefly, 10 μM EdU was administered to the complete culture medium during each medium exchange. After samples fixation, sectioning, dewaxing, and dehydration, sections were first permeabilized and blocked as described above. Sections were then incubated at room temperature for 30 min in a freshly prepared click chemistry cocktail, containing 6-FAM-Azide, protected from light. Following the incubation, the sections were washed three times with PBS + 3% BSA, and cell nuclei were counter-stained with DAPI as described above. After a series of washing steps, sections were further mounted using Fluoroshield mounting medium (Sigma Aldrich, USA) and coverslipped.

#### 2.8.4 TdT-mediated dUTP nick end labeling (TUNEL) staining and image analysis

TUNEL staining was performed using the ApopTag Fluorescein *In Situ* Apoptosis Detection Kit (Chemicon®, Merck KGaA, Germany) to assess cell death according to the manufacturer’s protocol. Briefly, fixed samples were first dewaxed and dehydrated using Xylol- Ethanol cassette. To permeabilize cells and facilitate reagent penetration, samples were incubated with a permeabilization solution containing 0.1% Triton X-100 in PBS for 10 min at room temperature. After another series of PBS washes, the TUNEL reaction mixture was prepared prior the usage according to the manufacturer’s instructions and applied to the samples. The samples were then incubated in a humidified chamber at 37 °C for 1 h in the dark. Following incubation, samples were washed three times with PBS to remove unbound reagent. For nuclear visualization, samples were counterstained with DAPI (1 µg/mL) for 15 min, followed by an additional PBS wash. Finally, samples were mounted using Fluoroshield mounting medium and coverslipped. Imaging was performed using a Leica THUNDER fluorescence microscope, utilizing the appropriate excitation and emission filters for both TUNEL and nuclear staining. Image acquisition settings were maintained constant among every sample for results consistency. To ensure assay specificity, positive (proteinase K-digested) and negative (without TdT) controls were included in each experiment. Stained slides were stored in the dark at 4 °C for short-term preservation prior to imaging.

Cell survival rate based on TUNEL staining was analysed using a custom algorithm programmed in IDL. Briefly, images were first converted to 16-bit (grey scale) and a universal threshold and a subsequent watershed function were applied to all images to split tightly co-localized nuclei into individual ones. Total cell number, based on DAPI staining (N_total_), and TUNEL-positive cells (N_TUNEL_) were counted using a connected component algorithm. The percentage of viable cells was calculated as[(N_total_–N_TUNEL_)/N_total_] x 100%. TUNEL-positive cells and DAPI-stained nuclei were colocalized using a masking function in order to split the DAPI channel into TUNEL-positive and TUNEL-negative cohorts.

#### 2.8.5 Microscopy data analysis

Analysis of the cut sections was performed using a Leica THUNDER Imager Live Cell & 3D Assay microscope, and image acquisition settings of each protein marker were maintained constant for results consistency. For each staining, the same slide number was selected for analysis, so we ensured the same depth of the section for each sample. 2 sections of each sample were analyzed. The sections were processed under standardized conditions in every experiment: control and experimental groups in each experiment were processed simultaneously and in a random order. To examine the specificity of staining, we performed the negative control (the same protocol without primary antibodies), which demonstrated no staining.

#### 2.8.6 Atomic force microscopy (AFM) imaging

AFM imaging was performed on tissue sections using NanoWizard Ultraspeed A (Bruker JPK, Germany) operating in contact mode as described by Andriotis et al. (2019). Sections mounted on glass slides were first dewaxed, rehydrated, and air-dried prior to imaging to ensure surface stability. For imaging, PNP-DB Cantilever A (NanoWorld, Switzerland) was brought into contact with the sample surface and imaging was performed at a setpoint of 2 nN. If not denoted differently, scans were acquired over scan areas of 5×5 µm at a scan rate of 1 Hz.

### 2.9 Mechanical characterization

#### 2.9.1 Micro-computed tomography (μCT)

Prior to tensile testing, micro-computed tomography (µCT) imaging was performed to obtain the geometry of the tendon constructs. For this, samples were mounted on a 3D-printed sample holder to maintain their form during imaging and for allowing to keep their bottom half in PBS to avoid dehydration while enabling enough contrast and sample hydration. The samples were placed in a sample holder of the device (µCT 100, Scanco, Switzerland). Imaging parameters were set to 45 kVp, 88 µA, with 250 projections per 180° rotation. Voxel resolution was set to 14.8 µm, and a 1.48 mm section of the construct in the linear region was scanned. Image reconstruction was carried out by manufacturer-provided software. Using medtool (medtool 4.8, Dr. Pahr Ingenieurs e.U., Austria) the image was further processed (Supplementary Figure S1, Supplementary Materials). A threshold was used to segment the sample from the background. If needed, the image was rotated to ensure alignment of the sample. The average cross-sectional area (CSA) of the segmented fibrin ring was then obtained by dividing its volume by the image height.

#### 2.9.2 Quasi-static tensile testing (pull-to-failure)

Mechanical testing of the tendon constructs was performed using a uniaxial universal testing device (zwickiLine Z2.5) (Zwick Roell, Germany) equipped with a high precision load cell (Xforce HP 50N, Zwick Roell, Germany). All tests were conducted at RT in hydrated conditions, with samples fully submerged in PBS to simulate physiological conditions. Samples were carefully mounted using custom-designed spools to ensure consistent alignment and prevent slippage. All tests were controlled and recorded using the manufacturer’s software (testXpert III, v1.9, Zwick Roell, Germany).

To assess the ultimate tensile properties, samples were subjected to a quasi-static uniaxial pull-to-failure test. Constructs were preloaded with a minimal force (10 mN) to eliminate slack and ensure a consistent starting length. Tensile loading was applied at a constant extension rate of 20 mm/min until rupture. Force-displacement data were recorded and converted into stress/strain curves based on the mean CSA determined for each experimental group by µCT. From these curves, the apparent tensile modulus (the slope of the curve) was derived using a custom algorithm, which selects the most linear region within a given strain range, based on the second derivative value.

### 2.10 Statistics

Statistical analysis of data was performed using GraphPad PRISM 8 software (GraphPad Software, Inc.). Results are presented as mean ± standard error of the mean (SEM). One-way analysis of variance (ANOVA) was performed in normally distributed populations, followed by Tukey’s post-hoc test for multiple comparisons, whereas the Kruskal-Wallis nonparametric test was performed for non-normally distributed data. Normality test was done using the Shapiro-Wilk and Kolmogorov-Smirnov tests. Differences between experimental groups were considered significant, with a confidence interval of 95% whenever p < 0.05 and are visualized as *,p < 0.01 as **, p < 0.001 as ***.

## 3 Results

### 3.1 Cyclic and static loading induce distinct gene expression profiles

It is known that cyclic mechanical loading is physiological for tendons, whereas static loading does not represent physiological conditions (Galloway et al., 2013; Pentzold and Wildemann, 2022). To obtain an overview of whether and how the mechanical stimulation regime (static vs. cyclic) affects the cells and their response to the loading, we performed an exploratory differential gene expression analysis using ONT sequencing, primarily intended to identify candidate pathways and transcriptional trends associated with the different loading conditions. PCA analysis demonstrated clear clustering of samples according to CL and SL, showing distinct transcriptomic differences. An average of 850k total reads per sample was obtained. Differential gene expression analysis identified 336 differentially expressed genes (DEGs) between CL and SL conditions, at a false discovery rate (FDR) threshold of <0.05. 294 genes were upregulated and 42 genes were downregulated in the CL group compared to SL.

The network analysis of DEGs revealed several tightly interconnected functional clusters (Figure 3B). One major cluster comprised pathways related to skeletal development and ECM organization, driven by structural genes such as *Col1a1, Col3a1* and *Sparc* (Figure 3A). In the CL group, we observed a significant upregulation of *Col1a1* and *Col3a1* - the key structural components of tendon ECM (Ansorge et al., 2011; Andarawis-Puri et al., 2015; Zhang et al., 2014), as well as enhanced expression of *Sparc*, a matricellular protein critically involved in tendon maturation and matrix organization (Gehwolf et al., 2016; Arai et al., 2024) (Figure 3A).

A second prominent cluster involved chemokine-mediated signaling and immune cell chemotaxis, coordinated by genes including *Cxcl1, Cxcl3, Cxcl6*, and *Ccl2*. We observed a slightly increased expression of inflammation-related genes in the SL group (Figure 3A). This data indicate a potential harmful effect of static loading on cells, leading to inflammation; however, this needs to be further investigated.

Smaller subnetworks were associated with cell proliferation, response to mechanical stimuli and response to nutrients.

### 3.2 Cyclic loading promoted tendon phenotype maintenance

**FIGURE 4:**
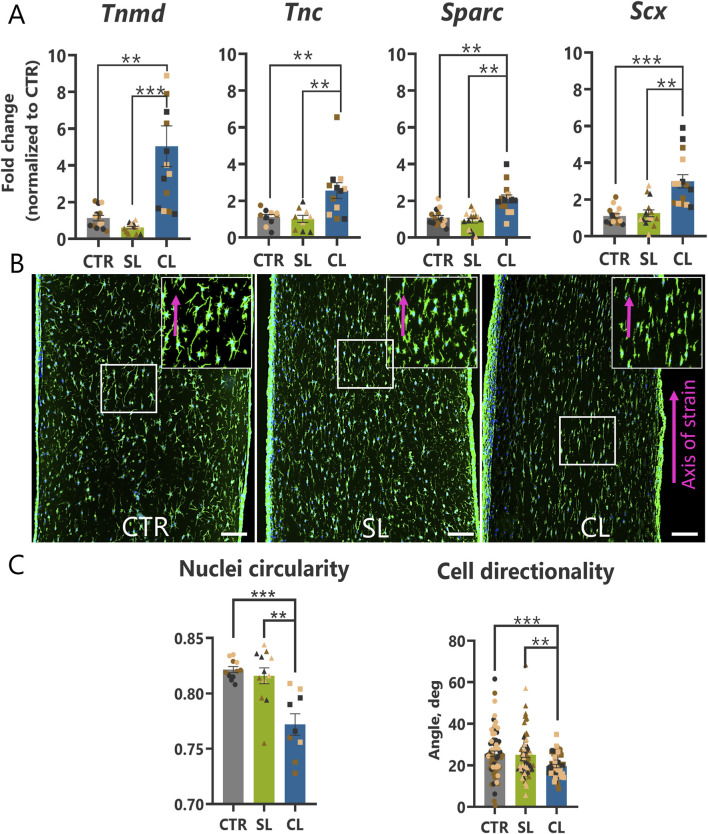
The effect of cyclic mechanical stimulation on phenotype maintenance and tendon-specific characteristics. **(A)** RT-qPCR analysis of *Tnmd, Tnc*, *Sparc* and *Scx* demonstrated increased expression in response to cyclic loading. Gene expression was normalized to *GAPDH* and is shown relative to gravity CTR: CTR–gravity control, SL - static loading, CL - cyclic loading. Data are shown as mean ± standard error of the mean (SEM), n=9-14 independent replicates from 3 experimental runs. Each color represents one experimental run. Statistical analysis was done with ordinary one-way ANOVA followed by Tukey’s post-hoc test for *Tnmd* and *Sparc* and nonparametric Kruskal-Wallis test followed by Dunn’s post-hoc test for *Tnc* and *Scx*. Values were considered statistically significant for p < 0.05. **(B)** Representative images of actin-FITC staining of a longitudinal section, demonstrating enhanced cell elongation along the loading axis upon cyclic loading. Scale bar = 250 μm. **(C)** Analysis of nuclei circularity and cell directionality. Data are presented as mean with standard error of mean (SEM). n=12 independent replicates (3 ROI per sample), 3 experimental runs (each represented by different color). Statistical analysis was done with an ordinary one-way ANOVA followed by Tukey’s post-hoc test.

It has been shown that mechanical stimulation is important for tendon-resident cells' phenotype maintenance (Kataoka et al., 2020; Pentzold and Wildemann, 2022; Wang et al., 2018b). To evaluate whether the type of mechanical stimulation affects phenotype maintenance or not, we performed RT-qPCR analysis of the key tendon-specific markers, namely *Tnmd, Tnc, Sparc,* and *Scx* (Wang et al., 2021; Huang et al., 2015). As shown in Figure 4A, CL and SL affect phenotype maintenance in a different manner. When CL was applied, we observed a statistically significant increase in *Tnmd, Tnc, Sparc,* and *Scx* expression compared to the SL and CTR groups, whereas no statistically significant differences were observed between the SL and CTR groups. Notably, CL led to a 4-fold increase in the expression of *Tnmd*, which indicates a positive effect of cyclic mechanical loading on tissue maturation (Docheva et al., 2005; Dex et al., 2017; Shukunami et al., 2018; Graça et al., 2022).

Another important tendon tissue characteristic is cell alignment. In our study, we use a heterogeneous population of cells, which we call TDCs. Both SL and CL groups demonstrated cell elongation along the axis of strain (Figure 4B), which indicates an effective differentiation of TDCs towards tenocytes (Li et al., 2021). Nevertheless, nucleus elongation was more pronounced in the CL group compared to the SL (circularity = 0.772 vs. 0.816, respectively) (Figure 4C). In addition, actin staining revealed a statistically significant difference in cell protrusion directionality, with more unified and greater elongation along the strain axis in cyclic vs. static loading (Figures 4B,C). Together, these results indicate superior phenotype maintenance in the constructs that underwent cyclic mechanical stimulation, indicating better tenocyte functioning compared to static mechanical stimulation.

### 3.3 Cyclic mechanical stimulation promoted the organization and remodeling of ECM

**FIGURE 5:**
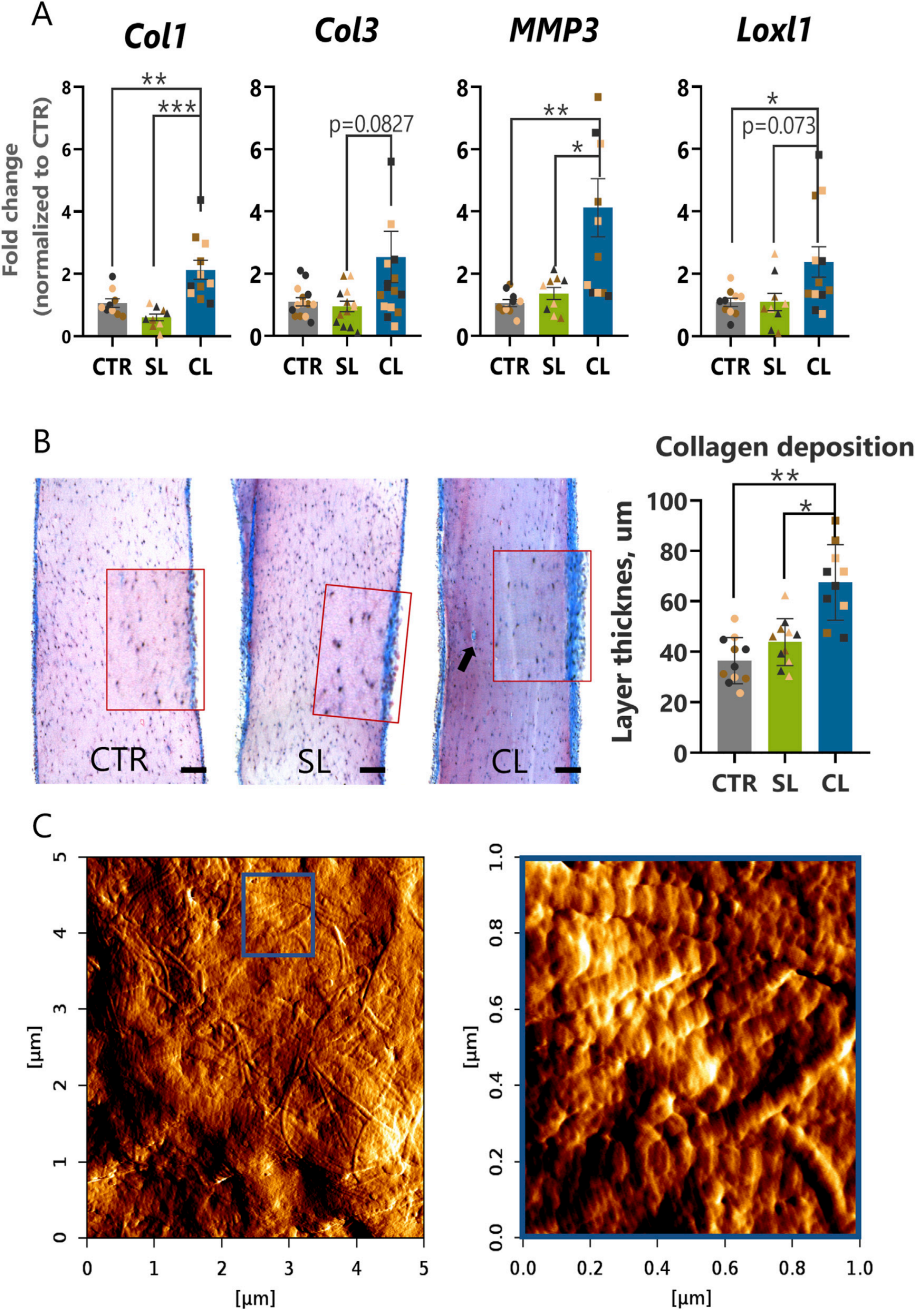
ECM remodeling upon different loading protocols. **(A)** RT-qPCR analysis of *Col1, Col3, MMP3* and *Loxl1* expression showed a significant increase in *Col1, MMP3* and *Loxl1* expression in the CL group. Analysis was done by calculating the ratio of these genes to *GAPDH*: CTR - gravity control, SL - static loading, CL - cyclic loading. Data are shown as mean with standard error of the mean (SEM), n=9-12 independent replicates from 3 experimental runs. Each color represents one experimental run. Statistical analysis was done with an ordinary one-way ANOVA test followed by Tukey’s post-hoc test. **(B)** Representative images of MSB staining and statistical analysis/quantification of collagen layer thickness, showing fibrin (purple/pink) replacement with collagen (blue) happening predominantly on the superficial layer. Scale bar = 200 μm. Statistical analysis was done using nonparametric Kruskal-Wallis test followed by Dunn’s post-hoc test, n=12 independent replicates from 3 experimental runs (each represented by different color). **(C)** AFM-based imaging of longitudinal section revealed collagen fibril formation upon CL. Collagen fibrils were identified by their characteristic D-periodicity (striped pattern).

Tissue composition can markedly affect tendon tissue performance. In this context, RT-PCR analysis of the genes involved in ECM remodeling showed statistically significant upregulation of *Col1,matrix-metalloproteinase3 (MMP3)*, and *lysyl oxidase homolog 1 (Loxl1)* expression in the CL group compared to both CTR and SL groups, with a 2-fold change for all three above-mentioned genes compared to SL, which is in agreement with the RNA-seq results (Figure 5A, Figure 3). We did not observe any significant differences in *Col3* expression, however, there was a trend for increased *Col3* expression upon cyclic stimulation, close to significance (p=0.0827).

We next investigated the localization of the freshly deposited ECM. MSB staining confirmed fibrin remodeling and replacement with collagen. In all experimental groups, collagen was predominantly deposited on the surface of the fibrin rings, as shown in Figure 5B in blue. In addition, the CL group showed some collagen deposition inside fibrin rings as indicated with a black arrow in Figure 5B. Notably, cyclic loading led to more collagen deposition compared to both SL and CTR groups, showing a 1.5-fold change in the thickness of the outer collagen layer. AFM imaging confirmed collagen fibril formation upon CL (Figure 5C) and identified by their characteristic D-periodicity (striped pattern). Imaging at random locations (n=8) confirmed fewer collagen fibrils formed in SL and CTR groups compared to CL.

### 3.4 TUNEL assay revealed improved cell survival in response to cyclic mechanical stimulation

**FIGURE 6:**
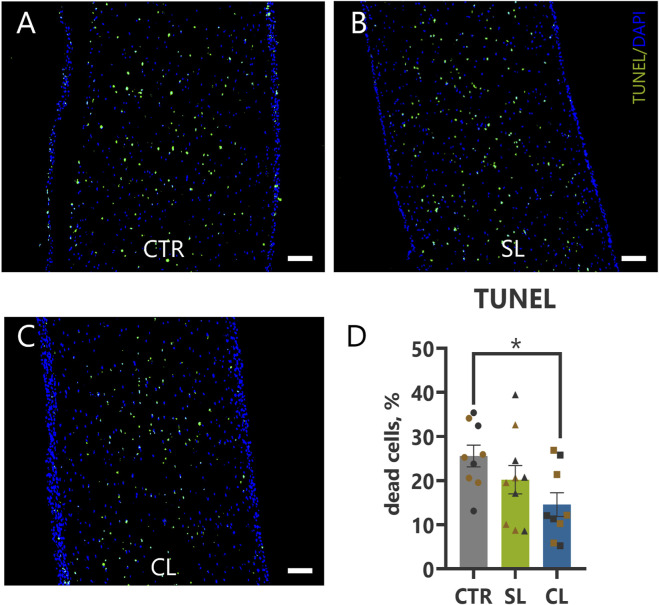
Analysis of apoptosis and cell death on longitudinal sections using TUNEL assay demonstrated higher cell survival upon cyclic stimulation. **(A–C)** representative images of TUNEL(green)/ DAPI (blue) staining, where CTR - gravity control, SL - static loading, CL - cyclic loading. Scale bar = 150 μm. **(D)** Percentage of dead cells, analyzed using a custom algorithm programmed in IDL (connected components: total number of cells (DAPI channel) and colocalized with TUNEL positive cells). Data are shown as mean with standard error of the mean (SEM), n=9 independent replicates from 2 experimental runs. Each color represents one experimental run. Statistical analysis was done with an ordinary one-way ANOVA test followed by Tukey’s post-hoc test.

To assess cell viability within the constructs, a TUNEL staining was performed. We observed significantly fewer dead cells upon cyclic mechanical stimulation compared to CTR (Figure 6). Overall average dead cell percentage in the three groups CL, SL and CTR was 13%, 20% and 25%, respectively. While in the CL group, dead cells were found mostly in the centre of the constructs, in the SL and CTR groups dead cells were observed throughout the cross-sections.

### 3.5 Cyclic loading promoted proliferation of TDCs

**FIGURE 7:**
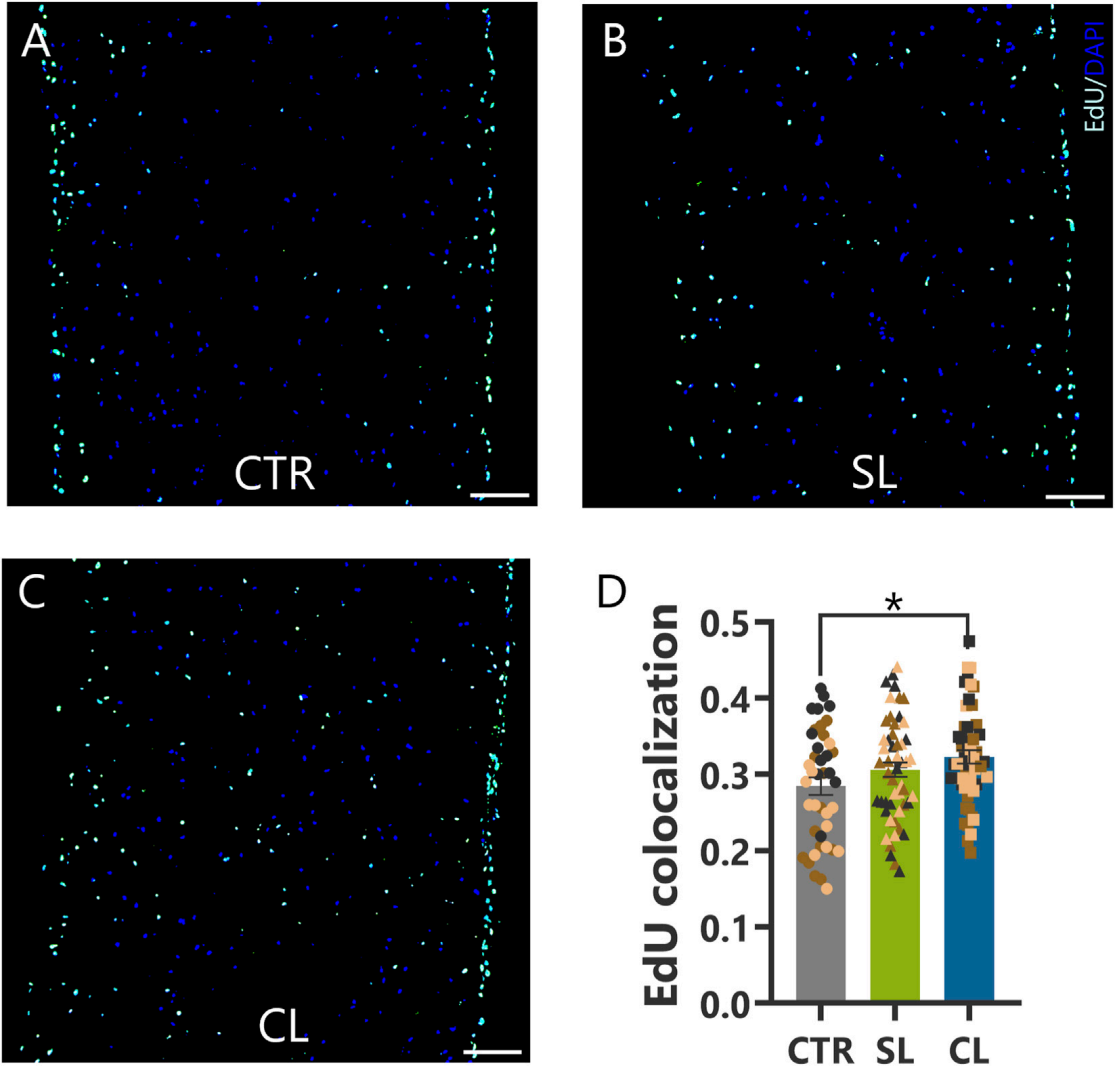
EdU click chemistry assay of longitudinal section shows greater proliferation upon CL. **(A–C)** Representative images show EdU-positive cells (green/turquoise) in the longitudinal sections. Cell nuclei were counterstained by DAPI (blue). CTR - gravity control, SL - static loading, CL - cyclic loading. Scale bar = 200 μm. **(D)** Co-localization analysis demonstrated more nuclei inside the constructs in the CL group compared to CTR, indicating more proliferative cells. The analysis was done using the Coloc2 plugin, FIJI. Data are presented as mean with standard error of the mean (SEM), n=12 (3 ROI per sample) independent replicates from 3 experimental runs. Each color represents one experimental run. Statistical analysis was done using nonparametric Kruskal-Wallis test followed by Dunn’s post-hoc test.

The EdU click chemistry assay was performed to assess cell proliferation under different mechanical stimulation regimes. Quantitative and qualitative analyses of EdU incorporation revealed a significant increase in proliferative activity among the experimental groups (Figure 7).

Spatial assessment of EdU-positive cells’ distribution demonstrated distinct proliferation patterns between groups. In the CTR group, proliferation was localized on the periphery (Figure 7A). In contrast, in both SL and CL groups (Figure 7B,C), proliferative cells were visualized throughout the constructs, with the CL group showing a more uniform and widespread EdU labelling. This spatial distribution pattern suggests improved nutrient perfusion, which resulted in greater cellular activity and proliferation upon cyclic mechanical stimulation. Qualitative analysis showed, that constructs subjected to CL exhibited a markedly higher number of EdU-positive nuclei compared with both the SL and CTR groups, indicating enhanced cell proliferation under dynamic stimulation (Figure 7D).

### 3.6 Cyclic loading significantly enhanced the stiffness of tissue-engineered tendon constructs

**FIGURE 8:**
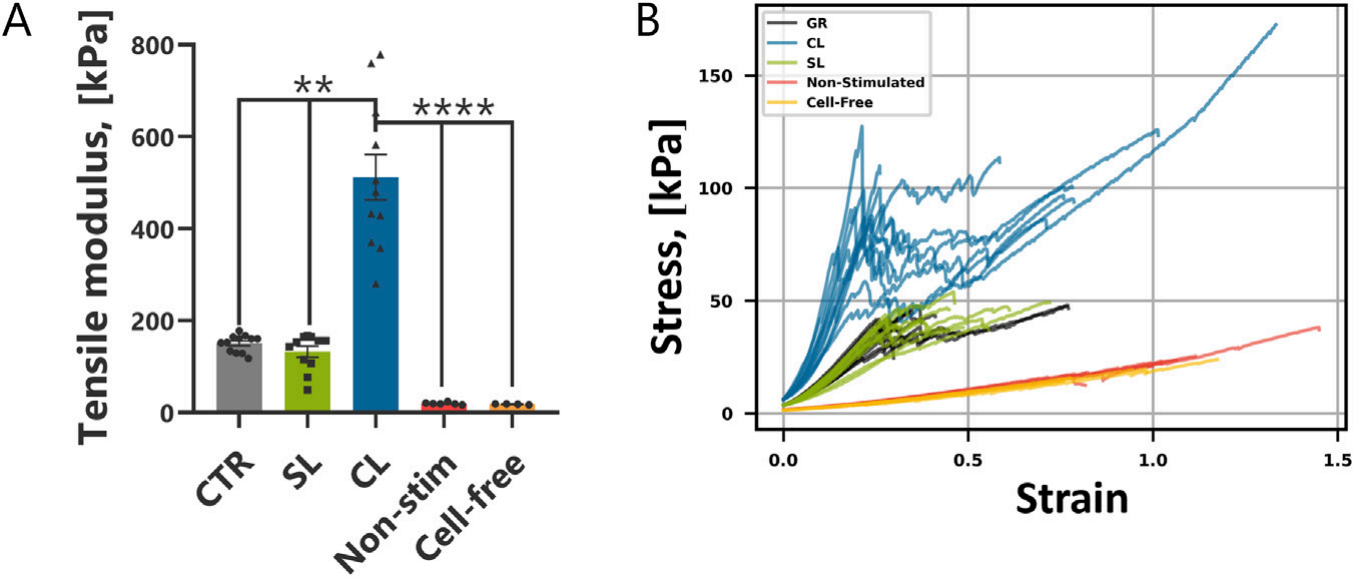
Mechanical testing of tendon constructs after 7-day stimulation shows increased stiffness upon CL. **(A)** Tensile moduli of the different groups were derived from the first linear region of each test with a linear fit to ensure a proper comparison. CTR - gravity control, SL - static loading, CL - cyclic loading, non-stim- cell-laden rings 2 days after casting, cell-free–rings without the cells. Data are shown as mean ± SEM, n=11 independent replicates from 2 experimental runs for CTR, SL and CL groups, n=4 independent replicates from 1 experimental run for cell-free group, n=independent replicates from 1 experimental run for non-stimulated group. Statistical analysis was done using nonparametric Kruskal-Wallis test followed by Dunn’s post-hoc test. **(B)** Representative stress-strain curves, showing non-linear mechanical response and delamination events upon cyclic stimulation.

To assess the mechanical functionality of produced tendon constructs, we performed uniaxial tensile testing of cultured fibrin rings after 7 day of mechanical stimulation. These experiments revealed a significantly (approx.3-fold) higher tensile modulus of the CL group compared to both SL and CTR groups (Figure 8A). This significant enhancement in mechanical stiffness is consistent with the observed improved ECM organization and collagen deposition. Furthermore, image analysis of µCT 3D datasets demonstrated a smaller CSA in the constructs subjected to CL relative to SL and CTR groups, suggesting matrix compaction and fiber alignment resulting from dynamic loading (Supplementary Figure S1).

In addition, the stress-strain curves of all groups exhibited a non-linear mechanical response, similar to the mechanical behavior of native tendon tissue at small physiological strains (Evans and Barbenel, 1975). It is noteworthy that during tensile testing of the constructs subjected to cyclic loading, signs of delamination events were observed close to the failure point (17%-20% strain), which appeared as multiple areas of stress drops in the stress-strain curves (Figure 8B). On the video records taken during tensile testing, this separation of ECM layers was visually confirmed by observing tiny threads separating from the construct, suggesting the coexistence of two structurally distinct matrix components: fibrin and newly synthesized collagen produced by TDCs (Supplementary Figure S2). This delamination phenomenon was exclusive to the CL group and was observed only at high strains close to the failure values. No delamination was observed during cyclic stimulation in the bioreactor.

This is in agreement with MSB staining, further confirming the presence of collagen mainly on the surface of the constructs, with greater layer thickness in the CL group (Figure 5B).

## 4 Discussion

This study aimed to investigate how cyclic and static mechanical stimulation influence tendon tissue formation in a fibrin ring system *in vitro*. By assessing biological, structural and mechanical parameters of constructs, we aimed to provide a more accurate and comprehensive answer to how loading regimes contribute to the functional development of tissue-engineered tendon constructs.

Our results show that cyclic loading has a positive effect on phenotype maintenance and ECM remodeling. This is shown in particular by the higher expression of ECM-related genes, namely *Col1*, as well as tenogenic markers, specifically *Tnmd*, upon cyclic mechanical stimulation compared to both, static stimulation and the unstimulated control group. These results are consistent with previous findings, showing that cyclic loading promotes a more mature tendon phenotype (Chen et al., 2024; Screen et al., 2005; Huisman et al., 2014; Breidenbach et al., 2015). Specifically, *Tnmd* has been shown to play an important role in the later stages of tendon maturation during development by regulating tenocyte proliferation and collagen fibril maturation (Docheva et al., 2005; Dex et al., 2017). The 4-fold increase in expression of *Tnmd* indicates a later and more mature stage of *in vitro* tendon development in CL in comparison to the other groups. Furthermore, recent studies on *Sparc* knock-out mice and TDSc, isolated from Achilles tendons of *Sparc* knock-out mice, have demonstrated the essential role of Sparc protein for regulating tendon tissue homeostasis and maturation by modulating the tissue mechanical properties and collagen fibrillogenesis (Wang et al., 2021; Gehwolf et al., 2016; Arai et al., 2024). Hence, the observed comparatively higher expression of *Sparc* upon cyclic mechanical stimulation further confirms the positive effect on the maturation of tissue-engineered tendons. This data is in agreement with AFM imaging results, confirming increased collagen fibril formation upon cyclic stimulation

In addition to biological markers, structural assessment can provide insight into tissue development and maturation. Here, the evaluation of the actin cytoskeleton staining further supported gene expression results, revealing enhanced cell alignment along the loading axis in the CL group. This is indicative of a more organized tissue architecture, a prerequisite for the development of functional tendon constructs. To identify cellular mechanisms underlying this response, it is important to consider the heterogeneity of tendon-derived cells (TDCs). These comprise several subtypes, including tendon stem and progenitor cells (TSPCs), tenoblasts and tenocytes, each with distinct roles and characteristics (Li et al., 2021). Based on the literature, mature tendon cell populations are typically dominated by tenocytes, which constitute approximately 90%-95% of tendon-resident cells, whereas TSPCs and tenoblasts constitute a smaller fraction, commonly estimated at approximately 5%-10% (Ruiz-Alonso et al., 2021; Buschmann and Meier Bürgisser, 2017). Tenocytes, as terminally differentiated tendon cells, play a vital role in secretion and building up the ECM, as well as in mechanotransduction. They are organized in a parallel alignment located between the collagen fibrils, with elongated cell nuclei and long cellular protrusions (Herchenhan et al., 2013).

As a result of the cell isolation method used, our constructs contained a heterogeneous cell population comprising the different tendon-resident cell types described above. Under cyclic mechanical loading, this population not only exhibited greater alignment along the deformation axis but also displayed distinct morphological adaptations, including fewer in number yet more elongated cellular protrusions, characteristic of terminally differentiated tenocytes. These observations may possibly reflect changes in cell population composition rather than true differentiation. However, it is important to note that all samples within a given experiment were cast with the cells derived from the same donor, the same isolation round and the same pooled cell suspension, with random allocation to experimental conditions. Therefore, we argue that the differences seen between groups in fact stem from the different mechanical stimulation protocols used. While we cannot completely rule out differences in cell population and cell subtype composition between samples the allocation process should even out such differences among the groups but could affect variability of results within groups. Finally, gene expression analyses, showing upregulation of the key tendon-specific markers upon CL, supports the idea that CL plays a crucial role in tenocyte differentiation and phenotype maintenance, thereby enhancing extracellular matrix (ECM) remodeling, which is in line with the greater collagen production found in the CL group. These results are in agreement with prior studies reporting the positive influence of cyclic mechanical stimulation on tenocyte differentiation (Burk et al., 2016; Wang et al., 2018b; Doroski et al., 2010; Gaut et al., 2020).

Results of the presented study demonstrate that cyclic mechanical stimulation exerts a beneficial influence on tenocyte functioning and tissue maturation *in vitro.* Although the present work did not directly assess intracellular signaling pathways, the observed enhancement in proliferative activity and accompanying upregulation of tendon-specific markers upon CL strongly imply that mechanotransduction mechanisms could be positively affected by CL. This interpretation is supported by previous research showing that focal adhesion kinase (FAK) phosphorylation is essential for the induction of tenogenic gene expression (e.g., *Scx, Tnmd* and *Col1a1*) and for differentiation in response to mechanical and growth factor stimulation in mesenchymal and tendon-derived cells in monolayer culture (Leahy et al., 2024). Furthermore, *Blache et al.* demonstrated that ERK1/2 phosphorylation was significantly increased in mechanically unloaded tendon fascicles, and its pharmacological inhibition prevented the increase in MMPs activity responsible for ECM degradation, thereby preserving mechanical integrity and suppressing pathological signs of remodeling (Blache et al., 2021). Taken these and our results together, we hypothesize that CL has a beneficial effect on mechanotransduction-mediated regulation of cell proliferation and ECM remodeling. Yet, this question is beyond the scope of the present work and requires further studies on the regulation of the signaling pathways, mentioned above, under different loading regimes. This will provide valuable insights into the molecular mechanisms underlying tendon regeneration.

Some of the beneficial effects of cyclic loading may partly arise from improved nutrient and metabolite transport within the three-dimensional constructs. Importantly, such enhanced mass transport is in itself a direct consequence of cyclic deformation and thus remains intrinsically linked to the applied mechanical stimulation regime. TUNEL staining revealed not only a smaller number of dead cells under cyclic mechanical stimulation but also a more spatially confined pattern of cell death. TUNEL-positive cells, representing both apoptotic and necrotic cells, were predominantly localized in the central regions of the 3D constructs subjected to CL, whereas the peripheral areas remained largely unaffected. This distribution likely reflects nutrient and oxygen gradients within the 3D matrix, as the geometry and thickness of the constructs can limit diffusion under static loading (SL) conditions, resulting in necrotic core formation (Place et al., 2017; LaBonia et al., 2016). In contrast, CL appears to create some media movements and enhance diffusion, increasing oxygen and nutrient delivery inside the construct, while simultaneously facilitating waste removal, which likely contributes to better cell survival. This is confirmed by the fact that the degree of cell death was significantly lower in the CL group than in the SL and CTR groups, where widespread cell death was observed.

During medium exchange, visual inspection confirmed the presence of media flow caused by the movement of the magnet. In the CL group, the medium had a uniform color, indicating effective mixing. In contrast, in both SL and CTR groups, we observed color stratification inside the falcon tube, specifically a paler zone in the central part, indicating local consumption of nutrients by cells in the absence of active mixing (Supplementary Figure S3, Supplementary Materials).

The MSB staining results further support this enhanced nutrient exchange and cellular activity upon cyclic stimulation, demonstrating more pronounced collagen deposition along the construct periphery, with thicker collagen layers. Such peripheral collagen deposition is indicative of active ECM remodeling by viable, metabolically active cells exposed to sufficient nutrient supply. In parallel, EdU incorporation assays demonstrated increased proliferative activity in the cyclic loading condition. Collectively, these findings align with previous reports demonstrating that cyclic mechanical stimulation promotes cell viability, proliferation and alignment of tenocytes (Burk et al., 2016; Sawadkar et al., 2020).

This is consistent with previous studies in which non-physiological mechanical stress induced inflammation and tissue degeneration (Gehwolf et al., 2025; Chen et al., 2024; Thorpe et al., 2015; Legerlotz et al., 2012). For example, *Chen et al.* investigated the impact of underload, normal load, and overload on oxygen-related reactions in tendon constructs. By applying cyclic mechanical stimulation at different strain levels, they observed that constructs subjected to normal loading exhibited higher mitochondrial content and more extensive mitochondrial networks compared to those exposed to under- or overload (Chen et al., 2024). In under- and overstimulated groups mitochondrial adaptation led to the release of extracellular mitochondria (ExtraMito) particles, which in turn promoted macrophage chemotaxis and enhanced the production of pro-inflammatory cytokines, including IL-6, CXCL1, and IL-18. In agreement with these observations, our RNA-seq analysis revealed increased expression of *Cxcl1, Cxcl3, Cxcl6, Ccl2,* and *Il6* under static loading conditions. The expression of these genes strongly implicate macrophage recruitment and inflammation. Taken together, these results suggest that the static mechanical stimulation in our study can be considered as a form of underload compared to the study of *Chen et al.*, while cyclic loading more closely resembles the normal mechanical conditions. Overlapping gene expression profiles between our static loading group and tendinopathic tendons strongly suggest that cyclic loading suppresses an inflammatory status in artificial tendon constructs (Chen et al., 2024).

Despite these promising findings, our study has several limitations. First, TDCs were obtained from a single donor, and therefore donor-to-donor variability could not be assessed. In addition, the population of TDCs was not characterized before constructs fabrication, limiting conclusions regarding the contribution of specific cell subpopulations to the observed response. The RNA sequencing sample size was limited (n=4 per group), and only one frequency and magnitude of mechanical strain were investigated.

Another important limitation concerns potential nutrient and oxygen transport constraints within the constructs due to their size, particularly under SL conditions. This may have contributed to cellular stress in the SL group and may therefore have influenced some of the observed differences between SL and CL constructs. Future studies should address this by improving nutrient supply during SL, for example by using dynamic perfusion, reduced construct thickness, modified sample geometry (hollow tube), or scaffold designs that facilitate diffusion.

Additionally, longer mechanical stimulation periods (14 and 21 days) may further improve construct maturation and functionality. Future work should also consider alternative or composite scaffold materials that better replicate native tendon mechanics.

One of the key findings of this study, reflecting all of the above-mentioned aspects, is an increase in the load-bearing capacity of tissue-engineered tendons upon cyclic stimulation. Here, cyclic stimulation outperformed static stimulation, with CL group showing approximately a three-fold higher tensile modulus compared to both SL and CTR groups. Although the absolute modulus values remain considerably lower than those reported for native tendons, which reach approximately 200 MPa after 7 days of postnatal development (Ansorge et al., 2011), our results represent early stages of functional properties that correspond to the progressive development of tendons *in vitro*. This underscores the critical role of cyclic mechanical stimulation in directing initial tissue organization and matrix remodeling, which are critical prerequisites for further subsequent maturation of tendon tissue.

From the tendon healing and regeneration perspective, these findings are in line with the well-established role of physiological mechanical stimulation in regulating cell alignment, matrix organisation, and controlled remodelling, whereas both mechanical deprivation and excessive loading are associated with impaired repair, persistent inflammation and degeneration (Gehwolf et al., 2025). In the *in vitro* model presented here, cyclic mechanical stimulation supported cell proliferation, upregulation of tenogenic markers, and enhanced ECM remodeling, thereby recapitulating key aspects of tendon mechanobiological environment during early tissue formation and tendon homeostasis.

To summarize, a favorable cellular response profile manifested by phenotype maintenance, increased expression of ECM proteins, and improved cellular processes (proliferation, cell death) supports the hypothesis that moderate cyclic loading promotes a balanced regenerative response and creates more favorable conditions for *in vitro* tendon tissue development.

## 5 Conclusion

This study demonstrates that dynamic mechanical loading provides a markedly superior microenvironment for tendon tissue maturation *in vitro* compared with static loading conditions. Cyclic stimulation enhanced cell viability, proliferation, and alignment, and upregulated tendon-specific markers such as *Scx, Tnc, Sparc, Tnmd*, and *Col1a1*, reflecting improved tenocytes functionality within fibrin scaffolds. Moreover, cyclic loading promoted ECM remodeling without eliciting inflammatory responses, thereby more closely mimicking the physiological conditions characteristic of tissue growth and adaptation.

Together, these findings emphasize the importance of biomechanical cues in regulating tenocyte behavior and ECM organization during early tendon development *in vitro* and highlight the necessity of incorporating controlled cyclic mechanical stimulation in *in vitro* tendon modeling to achieve more functional and biomimetic tissue constructs.

## Data Availability

The original contributions presented in the study are publicly available. This data can be found here: https://www.ncbi.nlm.nih.gov/biosample/54302253 https://www.ncbi.nlm.nih.gov/biosample/54302254 https://www.ncbi.nlm.nih.gov/biosample/54302255 https://www.ncbi.nlm.nih.gov/biosample/54302256.
