# Supplementary material for: Cyclic mechanical stimulation of TDCs improves tendon tissue maturation in vitro by enhancing collagen formation
Source: Front Bioeng Biotechnol. 2026 Jul 23;14:1773418. doi: 10.3389/fbioe.2026.1773418 (PMC13442637; doi:10.3389/fbioe.2026.1773418)
Supplement: Supplementary file 1 [file Table1.docx]

**Supplementary materials**

***Fig S1. Analysis of cross-sectional area (CSA) of the rings using μCT scan***

*a - µCT scan of a sample mounted onto a custom-made 3D printed ring holder with a wooden stick for μCT tube, b - transverse section of 1 mm height to scan on the SCANCO Medical software (μCT 100, SCANCO Medical AG), c- representative pictures of μCT scan for cyclic loading, static loading, and control groups. The thresholding and region selection were done on MedTool (medtool 4.8, Dr. Pahr Ingenieurs e.U., Austria). The volume calculation was done using Paraview (Paraview 5.13.2, Kitware, Inc.).*

***Fig S2. Delamination events, observed during tensile testing in the CL group***

***Fig S3. Effect of cyclic mechanical stimulation on the media flow***

***Fig S4. Heatmap of the combined 2 RNA-seq runs***
